# Current status and research progress of minimally invasive treatment of glioma

**DOI:** 10.3389/fonc.2024.1383958

**Published:** 2024-05-21

**Authors:** Hao Wu, Feng Zhou, Wenwen Gao, Peng Chen, Yao Wei, Fenglu Wang, Haikang Zhao

**Affiliations:** ^1^ Department of Neurosurgery, The Second Affiliated Hospital of Xi’an Medical University, Xi’an, China; ^2^ Department of Neurosurgery, The First Hospital of Yu Lin, Yulin, China

**Keywords:** glioma, minimally invasive treatment, photothermal therapy, photodynamic therapy, laser-induced thermal therapy, tumor treating fields

## Abstract

Glioma has a high malignant degree and poor prognosis, which seriously affects the prognosis of patients. Traditional treatment methods mainly include craniotomy tumor resection, postoperative radiotherapy and chemotherapy. Although above methods have achieved remarkable curative effect, they still have certain limitations and adverse reactions. With the introduction of the concept of minimally invasive surgery and its clinical application as well as the development and progress of imaging technology, minimally invasive treatment of glioma has become a research hotspot in the field of neuromedicine, including photothermal treatment, photodynamic therapy, laser-induced thermal theraphy and TT-Fields of tumor. These therapeutic methods possess the advantages of precision, minimally invasive, quick recovery and significant curative effect, and have been widely used in clinical practice. The purpose of this review is to introduce the progress of minimally invasive treatment of glioma in recent years and the achievements and prospects for the future.

## 1 Introduction

Among the primary malignant tumors of the central nervous system(CNS), neuroepithelial tumors of the brain are the most common. According to The Central Brain Tumor Registry of the United States (CBTRUS) Statistical Report: Primary Brain and Other Central Nervous System Tumors Diagnosed in the United States in 2016—2020,gliomas accounted for approximately 26.3% of all tumors. The most commonly occurring malignant brain and other CNS histopathology was glioblastoma(GBM)(14.2% of all tumors and 50.9% of all malignant tumors) (1). The treatment goal of glioma is to completely remove the tumor, effectively control the recurrence of the tumor, prolong the survival of the patient and enhance the living quality of patients Traditional therapeutic methods such as surgery, radiation and chemotherapy can reduce symptoms and prolong survival, but the therapeutic effect is limited by the malignant degree, growth site and molecular classification of glioma, and have certain limitations. In recent years, with the progress of science and technology, minimally invasive treatment has become the focus of glioma treatment and has been widely used in clinical practice. The new minimally invasive therapy has the advantages of precise targeting, reducing adverse reactions and complications. In addition, it has shown significant advantages in the treatment of tumors deep in the brain or in functional areas where surgery is difficult to reach.

Up to now, the main methods of minimally invasive therapy include photothermal therapy, photothermal therapy, laser-induced thermal therapy and the latest treatment strategies nano drug delivery system(NDDS) therapy and so on. In this article, we describe the therapeutic mechanisms and limitations of above approaches. Meanwhile, we will introduce the latest nanomaterial-based approaches for the diagnosis and therapy of glioma and provide new insights and references for future the glioma treatment.

## 2 Photothermal treatment of tumor

Photothermal therapy (PTT) is a treatment that utilize a material with a high conversion rate to inject it into the human body and convert light energy into heat energy under the irradiation of an external light source (generally near-infrared light) to kill cancer cells. Compared with traditional technology, the therapeutic effect of PTT only occurs at the tumor site, effectively avoiding the risk of killing normal cells and damaging the immune system, and it is a non-invasive and selective tumor treatment (2).

### 2.1 Mechanism and advantages of PTT

PTT is an emerging method to treat tumors by thermal ablation of tumor cells (3). During PTT, the temperature evolution at the tumor site is caused by the conversion of light energy into heat energy by a medium called a photosensitizer. In addition, the increased temperature can kill tumor cells while avoiding severe side effects on normal cells. The approach is effective, because tumor cells are less thermostability than normal cells. Specifically, the photosensitizer is first concentrated at the lesion site. The lesion is then irradiated with near-infrared light. Subsequently, the photosensitizer generates a lot of heat to ablate the tumor cells (4). Near-infrared light has been widely used in PTT because of its excellent tissue penetration and remote control (5). Furthermore, it possesses high resolution time and space adjustability, allowing for precise control (6).

**Figure 1:**
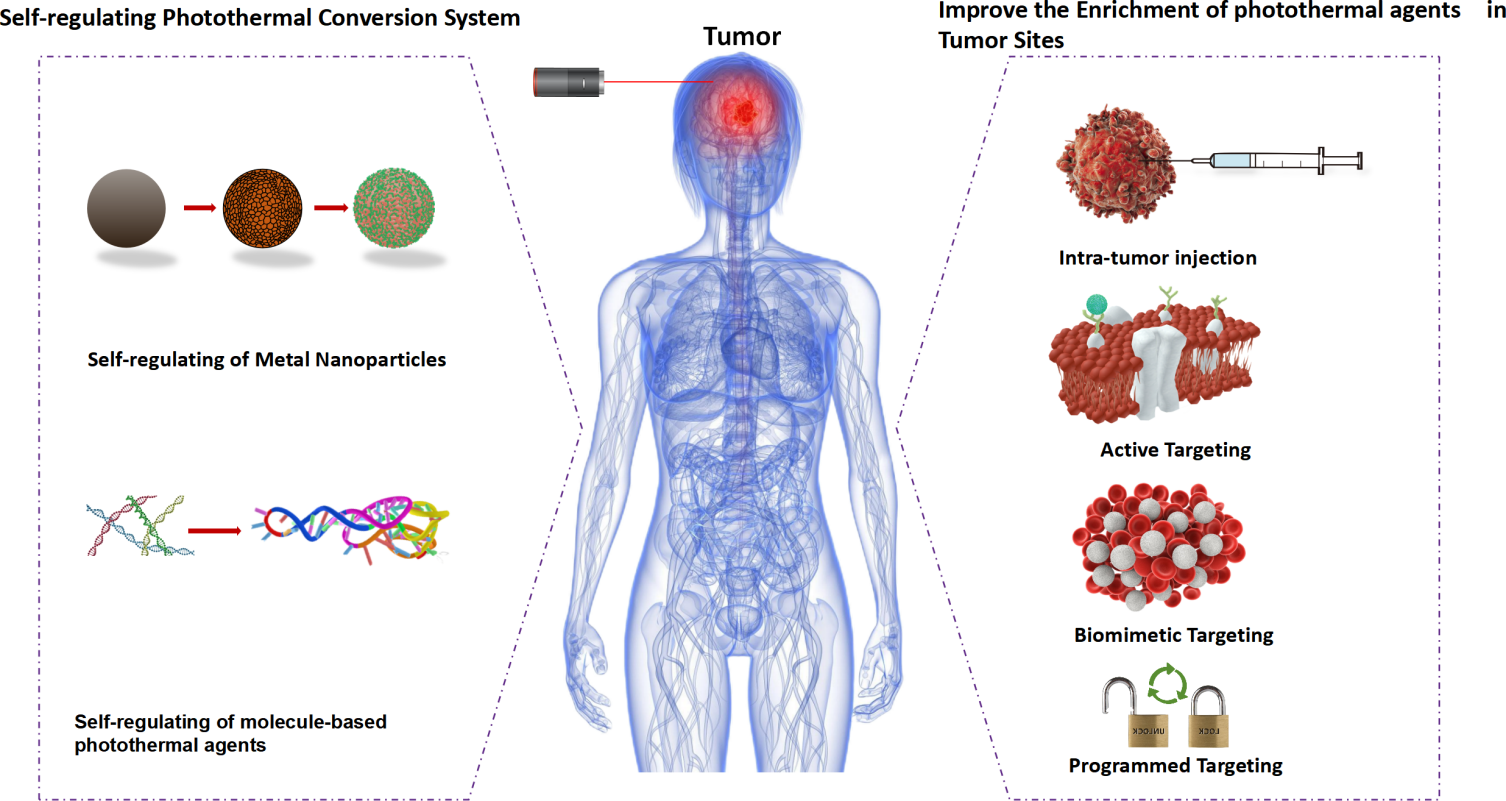
Schematic of strategies for improving selective photothermal therapy. photothermal agents (PTAs).

In order to explain the high photothermal conversion efficiency of photothermal materials doped with nanomaterials, it is necessary to understand the working principle of photothermal materials. As we all know, nanomaterials are typical mesoscopic systems with surface, small size and macroscopic quantum tunneling effects (7). At the same time, the optical, thermal, electrical, magnetic, mechanical and chemical properties of nanomaterials are significantly different from those of bulk solids (8). Previous studies have shown that metal-based, carbon-based and semiconductor-based nanomaterials can be used as photosensitizers in PTT systems. The reason for inorganic photosensitizers is that these materials have an optical phenomenon called local surface resonance (LSPR). After absorbing near infrared light, the electrons in the photosensitizer have obvious plasmon resonance effect. As a result, they can produce a significant thermal effect, heating the surrounding medium and making the temperature rise rapidly (9), which indicates that these nanomaterials are highly absorbent under near-infrared light. For example, Manikandan et al. studied the photothermal effects of platinum nanoparticles (Pt NPs) with a size between 5-6 nm (10). It was found that Pt NPs could increase the temperature by 9°C and could be further used as an ablative for photothermal ablation of Neuro 2A cells. In another study, Elbialy et al. developed multifunctional magnetic gold nanoparticles (Au NPs) with a diameter of 29 ± 4 nm, and confirmed that the prepared nanoparticles were effective as PTT drugs through histopathological and immunohistochemical studies (11) (**Figure 1**).

### 2.2 Research progress of PTT in glioma

The development of new diagnostic imaging and precision treatment methods for GBM is of great significance for improving the living quality and prolonging the overall survival of patients. He et al. successfully constructed a novel IR-II photoabsorbent conjugated polymer (PDTP-TBZ) from strong electron donor dithienopyrrole (DTP) and strong electron acceptor thiadiazole and benzotriazole (TBZ). Subsequently, c(RGDfK) cyclopeptide was modified on the surface of PT NPs to obtain a multifunctional nanodiagnostic reagent (cRGD@PT NPs) that can effectively target GBM neovasculature and tumor cells. Both *in vitro* and *in vivo* experiments shown that cRGD@PT NPs has high photothermal conversion efficiency and practical photoacoustic imaging ability under 1064 nm laser irradiation. The results of this work indicated that cRGD@PT NPs has great potential in highly efficient IR-II PTT guided by precise photoacoustic imaging(PAI), providing a good prospect for the treatment and diagnosis of GBM. At the cellular level, it has been proved that PDA@CUR NPs has the potential to leap-over blood-brain barrier(BBB) and can be rapidly taken up by brain glioma cells, and “CUR+ photothermal therapy” can effectively inhibit the proliferation of human and mouse brain glioma cells (12). Similarly, Sun et al. have developed a biomimetic nanoplatform AMNP@CLP@CCM for GBM targeted PTT and ICB(Immune checkpoint blockade) synergistic therapy. By loading the immune checkpoint inhibitor CLP002 into isomelanin nanoparticles (AMNPs) and then coating the cancer cell membrane (CCM).Due to the homing effect of CCM, the resulting AMNP@CLP@CCM can successfully cross the BBB and deliver CLP002 to GBM tissues. AMNPs, as a natural photothermal converting agent, is used for tumor PTT. PTT increases the local temperature, not only enhances the penetration of the BBB, but also upregulates the PD-L1 level of GBM cells. Importantly, PTT can effectively stimulate immunogenic cell death, induce tumor-associated antigen exposure, and promote T lymphocyte infiltration, thereby further enhancing the anti-tumor immune response of GBM cells to CLP002-mediated ICB treatment, thus significantly inhibiting the growth of GBM in situ. Therefore, AMNP@CLP@CCM has great potential in the synergistic treatment of *in situ* GBM by PTT and ICB (13).

At present, with the rapid development of nanotechnology, photothermal therapy has achieved fruitful achievements in the treatment of glioma, but it only stays in the cell or mouse test stage, and has not really entered the clinical trial stage. We hope that more in-depth research can be combined with clinical cases to improve the prognosis of patients and improve the survival of patients. There is still a long way to go, but we firmly believe that through the efforts of generations of scientists and we will ultimately be able to overcome this global problem.

### 2.3 Limitations of PTT

In the near future, PTT will continue to play an important role in clinical applications and will require a lot of effort in related scientific research. First of all, the physical and chemical modifications make the photosensitizer have high photothermal conversion efficiency and good biocompatibility. Second, light-driven nanomaterials facilitate fast, remote control and tunable movement of NPs. Third, PTT combined with other tumor therapies effectively excised tumor cells without seriously damaging adjacent normal tissue. In addition, designing dual-acting tumor therapies is critical for multiple functions such as drug delivery, real-time imaging, and chemical-PTT. Despite impressive progress in developing photothermal nanomaterials, many challenges remain in terms of clinical application. Biocompatibility, long-term toxicity, dose-dependent toxicity, targeting specificity, and biodegradation are still need to be solved. It is important to note that the potential threat of photosensitizers to patients and the environment cannot be ignored. In practice, this review provides valuable information for the preparation of novel photosensitizers and will motivate researchers to invest more effort in PTT methods.

## 3 Photodynamics Therapy

PDT is a modern, non-invasive therapy for the treatment of non-oncologic diseases as well as various types and sites of tumor. It is based on the topical or systematic application of photosensitive compounds-photosensitizers, which are accumulated in pathological tissues. The photosensitizer molecule absorbs the appropriate wavelength of light and initiates the activation process, resulting in the selective destruction of inappropriate cells. Phototoxic reactions occur only within the pathological tissue, in the region where the photosensitizer is distributed, making selective destruction is possible. Over the past decade, the development of nanotechnology has accelerated significantly. The combination of photosensitizers and nanomaterials can enhance the efficiency of PDT and eliminate its side effects. The use of nanoparticles enables a targeted approach that focuses on specific receptors, and therefore, increases the selectivity of photodynamic therapy. This section will briefly describe the anti-cancer application of PDT, its advantages and possible modifications to enhance its effects (14).

### 3.1 Mechanism of PDT treatment

**Figure 2:**
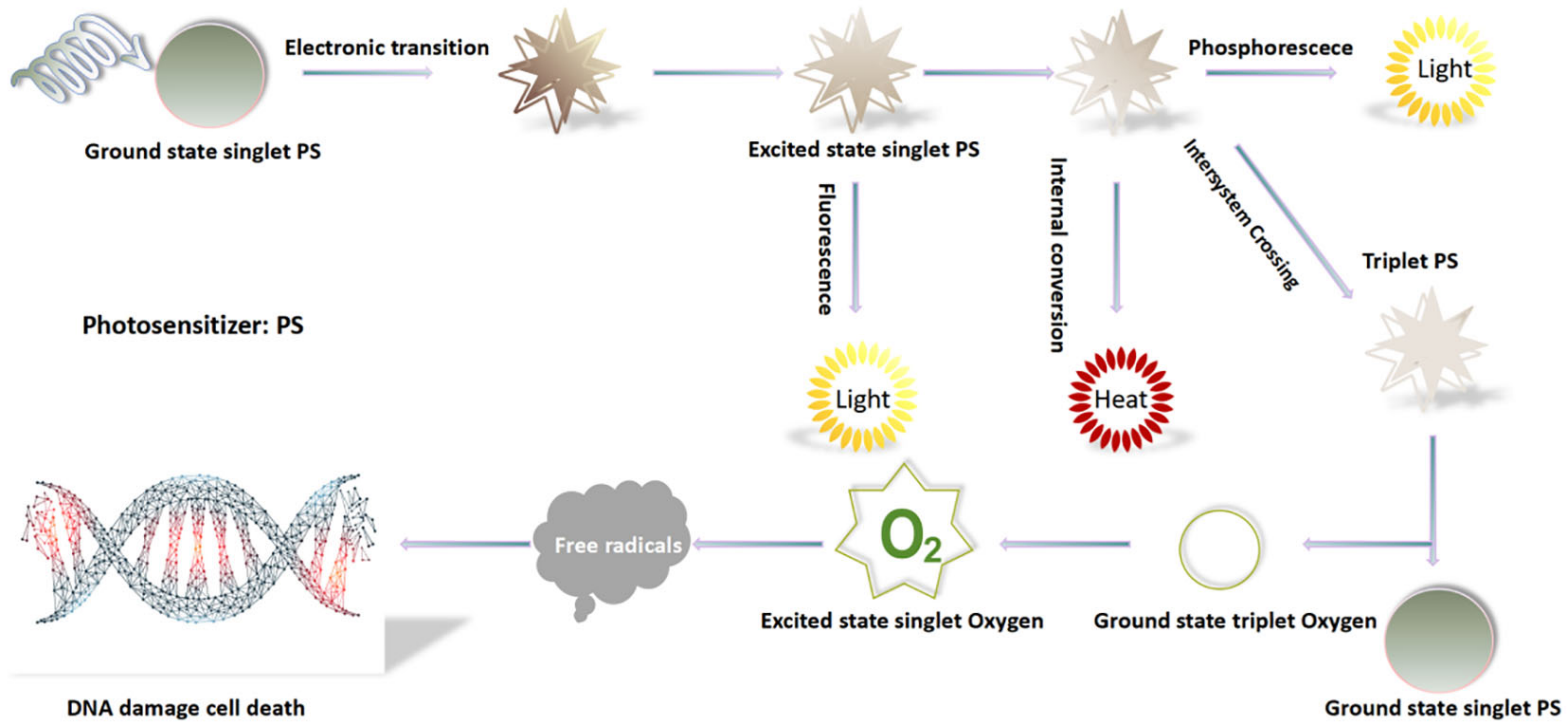
Mechanism of PDT treatment.

Molecular mechanism of PDT is based on the three non-toxic components, which produce the desired effects within pathological tissues only by mutual interactions between: There are two main mechanisms of the photodynamic reaction. Both are closely dependent on oxygen molecules inside cells. The first stage of both mechanisms is similar. A photosensitizer, after entering the cell, is irradiated with a light wavelength coinciding with the PS absorption spectrum and is converted from the singlet basic energy state S° into the excited singlet state S1 because of the photon absorption. Part of the energy is radiated in the form of a quantum of fluorescence, and the remaining energy directs a photosensitizer molecule to the excited triplet state T1-the proper, therapeutic form of the compound (**Figure 2**) (15).

### 3.2 Research progress of PDT in treatment of glioma

Using photodynamic technology to treat tumor, light source is needed to activate high concentration of photosensitizers in tumor tissue to produce functional oxygen source-mono-linear oxygen. The power of light source is directly related to the killing effect of tumor and the damage of normal tissue (16). Photosensitizers have been shown to accumulate within tumor cells, and PDT targets malignant tumor cells to exert cytotoxic effects. As an auxiliary means of surgical treatment, PDT can effectively inactivate tumor cells and kill residual tumor cells around tumor lesions, which is a feasible treatment plan for brain tumors. In a recent single-center, non-randomized phase I/II clinical study, researchers evaluated the feasibility of PDT in the treatment of malignant brain tumors in children and adolescents. The main key points were the safety of PDT treatment (phase I) and overall survival after PDT (OS, phase II), and the secondary key point was PFS after PDT (17). The pathological findings of the included patients included intracranial stromal tumor, stromal astrocytoma, diffuse midline glioma carrying H3K27M mutation, glioblastoma, and pediatric high-grade glioma, with OS and PFS acquired at 21 months and 6 months, respectively. However, the clinical study included few cases and could not obtain a definitive conclusion on PDT. At the same time, another team used interstitial photodynamic iPDT technology to treat newly diagnosed glioma patients, and the results showed that PFS was 16.4 months and OS was 28.0 months, but the study limited the tumor volume of included patients and selected small tumor volume (diameter<4 cm), thereby reducing the risk of harm due to edema (18). Compared with the classical treatment of glioma, PDT significantly enhanced the overall survival and progression-free survival of patients. However, the data published by different teams did not contain the molecular typing of glioma, such as MGMT methylation information, so the effect of photodynamic therapy on different grades of glioma needs to be studied in multi-center and large samples. In the past few decades, PDT for aggressive tumors of the central nervous system has achieved good clinical results, but there is no consensus among different centers on standardized treatment. The selection of photosensitizers and light source parameters in the large center studies of central nervous system photodynamic therapy in the world are inconsistent. The third generation of photosensitizers developed at this stage can be applied to clinical applications, which will greatly improve the targeting of photodynamics. Combined with optical fiber devices with efficient transmission, it is believed that photodynamics can benefit more patients with aggressive brain tumors, especially GBM patients.

### 3.3 Limitations of PDT

Although the clinical treatment of PDT has achieved remarkable success, its wide clinical application is limited by the obvious phototoxicity of traditional phototherapy (19). The main cause of phototoxicity is the uncontrolled distribution of photosensitizers, which when exposed to natural light can lead to untargeted effects in normal tissues, including skin, blood vessels and liver, resulting in damage to normal cells. Moreover, the irregular distribution of photosensitizers may result in low accumulation in tumor cells, limiting the efficacy of PDT. From a technical point of view, selective irradiation of tumor cells is a challenge that requires the development of a method that can deliver a controlled and sustained delivery of photosensitizers directly to the tumor site. Furthermore, the PDT treatment area requires more oxygen to obtain oxygen free radicals to kill the tumor cell, but the tumor is in a state of high oxygen consumption, which may further impact the therapeutic effect.

## 4 Laser-induced thermal therapy of tumor

**Figure 3:**
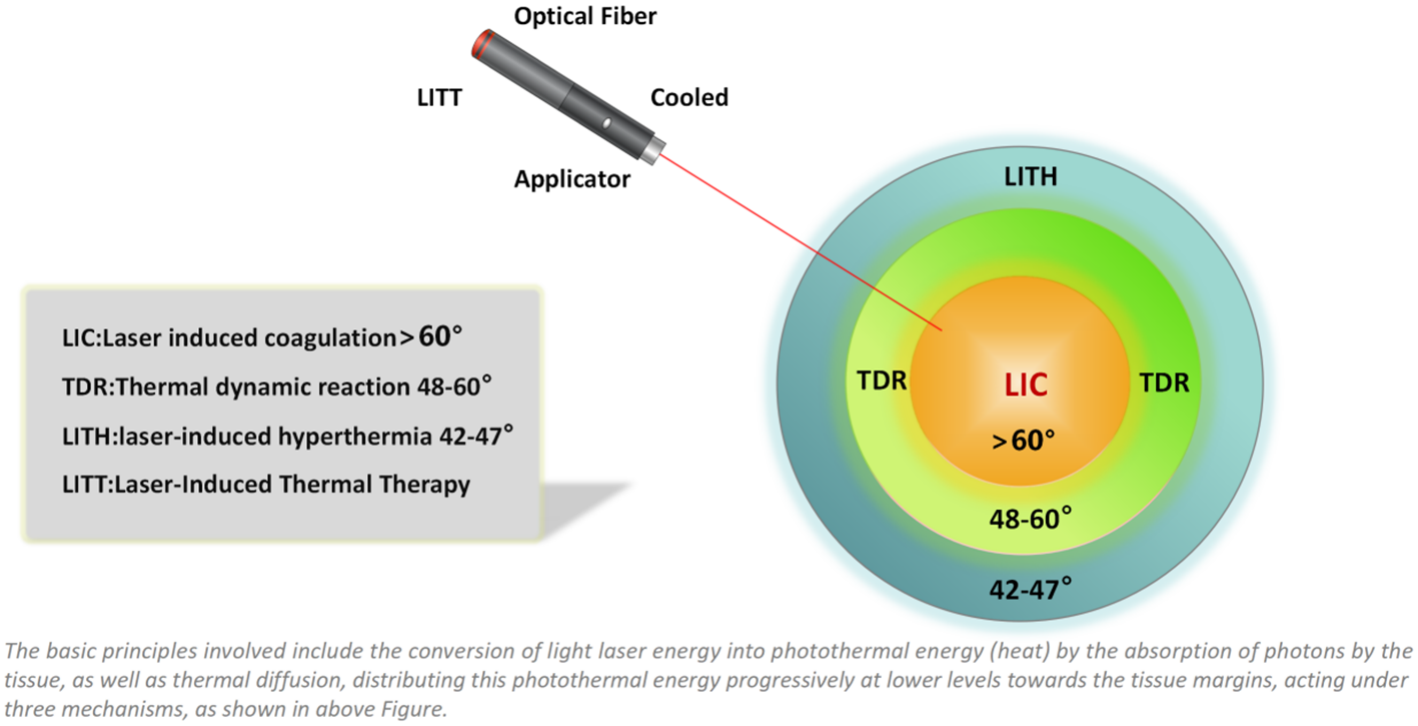
Graphic representation of photothermal mechanisms in laser-induced thermal therapy.

**Figure 4:**
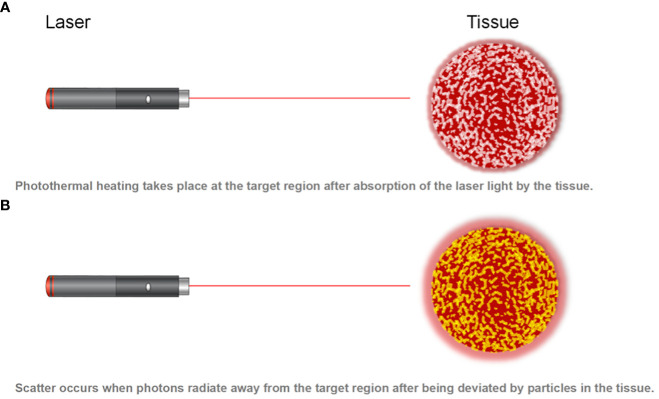
Photon absorption and scatter.

Laser-induced thermal therapy (LITT) is a minimally invasive surgical approach based on thermal ablation provided by laser via flexible conductive fibers, acting by external or interstitial radiation (20, 21) (**Figures 3**, **4**). During the last 30 years, LITT has gained attention in various clinical scenarios, such as liver cancer, lung cancer, brain tumors, and recurrent or advanced head and neck tumors, among others. Since its creation in 1983, there have been technical improvements to increase its safety and precision, especially with advances in magnetic resonance (MR)-guided therapy (22). The basic principles involved including the conversion of light laser energy into photothermal energy (heat) by the absorption of photons by the tissue, as well as thermal diffusion, distributing this photothermal energy progressively at lower levels towards the tissue margins, acting under three mechanisms, as shown in **Figure 4**: laser-induced coagulation (LIC: > 60°C), dynamic thermal reaction (TDR: 48–60°C) and laser-induced hyperthermia (LIHT: 42–47 °C). In the core of the irradiated area, there is virtually instantaneous irreversible cell destruction at temperatures > 60 °C,while the tissue margins may suffer reversible cell damage (42–60°C), and, in the case of tumors, it becomes a region with a high rate of relapses, acting better in conjunction with chemotherapy (23, 24).

LITT has been used for many years as a minimally invasive treatment for brain metastases, epilepsy, necrosis, and glioma. With the improvement of thermal monitoring and ablation accuracy, especially the application of MR thermal imaging technology in surgery, and now the emergence of two commercial laser systems, LITT is gradually being accepted by more neurosurgical centers. In recent years, several new concepts for glioma treatment have been proposed and are being investigated, such as adjuvant chemotherapy or radiotherapy after LITT, immunotherapy and LITT combination therapy. The purpose of this study was to summarize the development of LITT, especially brain gliomas and possible future prospects.

One interesting possible indication is to use the disruption of the BBB after LITT to make adjuvant chemotherapy more effective. It has been reported that the effects of BBB disruption after LITT can be demonstrated radiologically by enhanced peripheral contrast (25, 26). Recently, Leuthardtet et al. reported that by detecting serum specific enolase levels, LITt-induced destruction of the peritumoral blood-brain barrier reached its peak at around 3 weeks and lasted for about 4-6 weeks (27).

Although there is no direct evidence (such as case-control studies) to support LITt-induced BBB disruption leading to better outcomes. Carpentier et al. speculated that LITT opening of BBB rather than local control improves survival in patients with recurrent GBM (28). LITT has become an alternative to surgical resection in the treatment of gliomas. However, treatment outcomes for isocitrate dehydrogenase 1 and 2 (IDH1/2) mutant gliomas have not been reported. Johnson’s study described a single-institution cohort of patients with grade 2/3 glioma with IDH1/2 mutations receiving LITT. They collected data on patient presentation, radiological characteristics, tumor molecular profiles, complications, and outcomes. We calculated progression-free survival (PFS) and tested factors that were significantly associated with longer PFS. Overall, progression occurred in 22.7% of the cohort during a median follow-up of 1.8 years. The 3-year and 5-year PFS are estimated to be 72.5% and 54.4%, respectively. This is the first study to investigate the prognosis of patients with IDH1/2 mutated glioma after LITT. Our findings suggest that LITT is an effective option for the treatment of IDH1/2 mutant gliomas (21). It has also been reported that LITT-induced hyperthermia may have a synergistic effect with ionizing radiation, or may disrupt the BBB and facilitate the delivery of chemotherapy (23, 29).

LITT is complementary to surgical resection, radiation therapy, oncology treatment areas, and systemic therapy, and is especially suitable for patients at high risk of surgical resection due to tumors located in good areas or poor functional status. The increased incidence of cerebral edema after LITT compared to surgical resection must be balanced against these factors. LITT has also been shown to induce transient disruption of the BBB, particularly in the area surrounding the tumor, which allows enhanced central nervous system delivery of anti-tumor drugs, thus greatly expanding the Arsenal against brain tumors, including highly effective anti-tumor drugs with low BBB penetration. In addition, heat-induced immunogenic cell death is another secondary side effect of LITT, which makes immunotherapy an attractive adjunct treatment for brain tumors. Many large studies have demonstrated the safety and efficacy of LITT in the treatment of various CNS tumors, and as the literature on this new technology continues to grow, so will its indications (30).

### 4.1 Mechanism and advantages of LITT

One of LITT’s features is its real-time thermal monitoring capability. Researchers McNichols et al. used LITT to treat lesions in the brains of dogs and pigs, and controlled the process of thermal energy and laser ablation through the feedback mechanism based on precise MRI positioning (31). The system effectively regulates heat, eliminates carbonization and evaporation, while protecting the laser’s fiber optic attachment. MRI image results can also provide important information such as tumor blood supply and provide a more comprehensive reference for surgery. The compatibility of LITT with real-time MRI temperature measurement ensures the safety and homogeneous management of surgery, thus increasing the efficacy of this method in the treatment of intracranial lesions (32–34). Compared with traditional surgical resection, LITT is less traumatic, only needs to enter the deep brain through the small hole in the scalp for treatment, and can be used repeatedly without worrying about dose toxicity (such as radiation therapy) or drug resistance (such as chemotherapy). At the same time, LITT can destroy BBB. It can also increase the permeability of therapeutic drugs (27, 29, 35). Muir et al. ‘s research results showed that patients who received LITT multiple times for the treatment of recurrent GBM could also tolerate it well, effectively extending the survival time and enhancing living quality of patients (36).

### 4.2 Research progress of LITT in glioma

The standard treatment for newly diagnosed high-grade glioma(HGG) patients is maximum safe resection followed by chemoradiotherapy. In some cases, this standard strategy cannot be employed when the tumor involves an important or hard-to-access area due to an unacceptable risk of morbidity. In these patients, the standard treatment includes biopsies and chemoradiotherapy, which is unfavorable in terms of tumor cell reduction. Mohammadi et al. demonstrated improvements in disease-specific OS and progression-free survival(PFS) in patients receiving pre-LITT post-chemoradiotherapy compared to controls matched by propensity scores based on age, sex, tumor location, and tumor volume. This control group, from other institutions that did not use LITT for this patient population, received only biopsials followed by chemoradiotherapy. These authors also demonstrated that ablation degree is an independent predictor of disease-specific OS and PFS (37).

Bilateral/butterfly glioblastoma (bGBM) has a poor prognosis. Resection of these tumors is limited due to severe comorbidities resulting from surgery. LITT offers a minimally invasive cell reduction therapy for deep tumors such as bGBM. The objective of the study was to evaluate the safety of bilateral LITT in patients with bGBM. A total of 25 patients were included. Fourteen patients underwent biopsy only, and 11 underwent biopsy+LITT(7 bilateral LITT, 4 unilateral LITT). No intraoperative or postoperative complications occurred in the treatment group (0%). Tumor volume was negatively correlated with treatment scope (r2 = 0.44, *P* = 0.027). Median progression-free survival was 2.8 months in the biopsie-only group and 5.5 months in the biopsy + LITT group (*P* = 0.026). Median overall survival was 4.3 months in the biopsy alone group and 10.3 months in the biopsy + LITT group (*P* = 0.035). Bilateral LITT for bGBM can be performed safely and shows early improvements in progression-free survival and long-term survival outcomes in these patients (38). A recent meta-analysis reported the use of LITT in newly diagnosed and relapsed high-grade gliomas. The results are similar to those reported in previous literature, demonstrating the benefit of LITT in OS and PFS as long as more than 95% of tumors are removed. LITT seems to be a reasonable option for patients with deep, hard-to-access, or vital functional tumors. Using the technique, the number of cells in this type of tumor can be reduced with minimal brain manipulation and a complication rate comparable to that of craniotomy. Similar to surgery, in order to obtain meaningful survival benefits, tumors should be ablated by at least 78% to 80% (39). Beaumont et al. indicated that the median survival of LITT treatment was 7 months in patients with corpus callosum HGG, compared with surgical resection of about 65%. In this study, the tumors were larger (≥Patients with 15 cm^3^) were 6 times more likely to develop complications (40).

### 4.3 Limitations of LITT therapy

LITT plays an increasingly important role in the treatment of brain glioma, but it also has certain limitations and shortcomings. (1) Indications: LITT is usually suitable for small tumors (including butterfly GBM), and for large tumors or tumors with obvious cystic degeneration, the therapeutic effect needs to be further improved. (2) Imaging: MRI-guided LITT needs to pay close attention to the shape and location of the tumor during surgery. However, the resolution and imaging depth of MRI are limited, which may not clearly show the edge of the tumor or the boundary between the tumor and the surrounding tissue, nor can it visually observe the bleeding during surgery, which may increase the risk of surgery. (3) Ablation scope: The size and energy limitations of the therapeutic equipment used in LITT may not ablate the entire tumor, leading to an increased risk of recurrence. (4) Surgical operation: LITT requires high skills and experience of doctors, otherwise it may lead to surgical failure or serious complications. During the operation, the device must be guided through the skull into the brain, which is a complicated process, and improper operation may increase the operation time and the risk of bleeding.

To sum up, LITT is a minimally invasive procedure with a lower complication rate compared to craniotomy. The most common complication of LITT is neurological dysfunction, with temporary disorders ranging from 0-29.4% to permanent disorders ranging from 0-10%. This is associated with direct white matter damage caused by heat, leading to permanent impairment, and temporary impairment caused by white matter tract displacement or edema. Bleeding complications may also occur within the tumor area or locus. Intractable cerebral edema is also associated with laser ablation of larger tumors. Recent observations suggest that these large lesions may require immediate surgical removal. Another rare complication is pseudoaneurysm formation and rupture, which appears to be related to heat damage to large and medium-sized brain arteries. Careful planning before surgery using MRI angiography or catheterization can increase the safety of surgery. Other minor complications, including infections or wound problems, are less common than open surgery because of the advantage of having a smaller skin wound. Designation: Newly diagnosed HGG: Small deep brain tumors (including butterfly-shaped gliomas), open surgery may have a high risk of complications, and patient preference. Recurrent HGG: Small or nodular recurrence. For larger relapses, LITT may have advantages over craniotomy because the incision on the irradiated scalp is minimally invasive and small in size. Whether complete or near-complete ablation of one or both trajectories is feasible for newly diagnosed or relapsed HGG. The benefits of partial ablation in OS and PFS appear to be limited. For larger tumors, LITT may need to be combined with immediate surgical removal. However, although the procedure is more convenient due to the vascular less nature of the tissue and can be performed with minimal craniotomy, this approach does result in longer operation times for these combined procedures. Radiosurgical resistant metastases. Drug-resistant radionecrosis (RN) as a second-line treatment option. The volume is usually less than 40-50 ml.

## 5 Tumor treating fields

TT-Fields is a new treatment method. As a selective electric field, it interrupts cell division by generating a low-intensity medium-frequency selective electric field around the tumor, and then kills tumor cells. It can achieve the effect of cancer treatment at the frequency of 200kHz. Studies have shown that TT-Fields can prolong the survival of GBM patients (41). TT-Fields was approved by the FDA in 2011 for the treatment of recurrent GBM and in 2015 for the treatment of newly diagnosed GBM. According to the 2017 Guidelines for Central nervous System Tumors in the United States, TT-Fields can be used in GBM with KPS≥60 and MGMT promoter methylation or non-methylation. After receiving standard concurrent chemoradiotherapy, Temozolomide combined with TT-Fields is recommended for patients aged ≤70 years. TT-Fields may also be used in patients aged 70 who receive non-large frit radiotherapy,i.e.,temozolomide adjuvant chemotherapy, which is standard concurrent chemoradiotherapy. TT-Fields may be considered for recurrent GBM, whether diffuse, multiple, locally resectable or unresectable (42).

### 5.1 Treatment mechanism of TTfields

**Figure 5:**
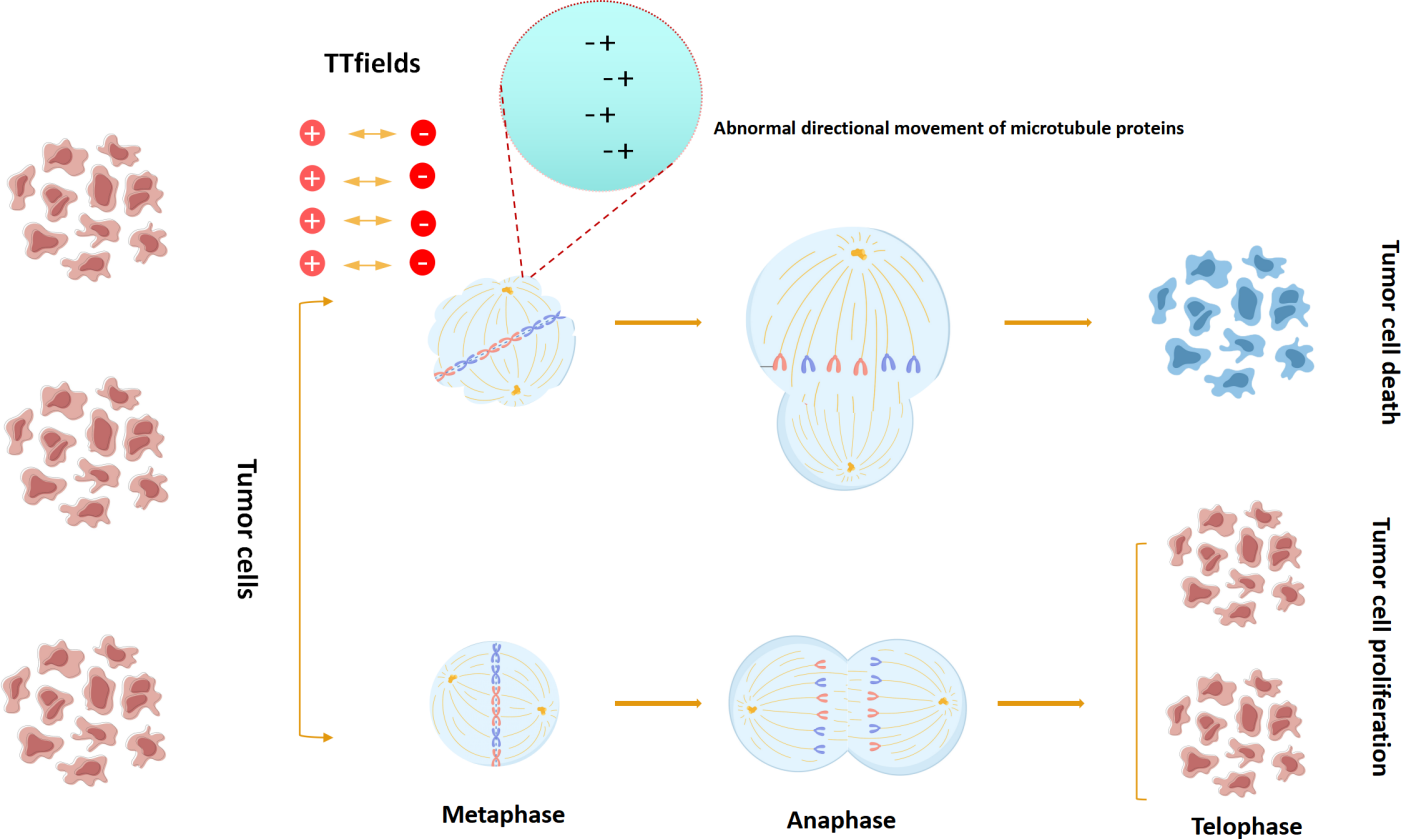
TTFields model for interfering with tumor cell mitosis.(In the anaphase of tumor cell mitosis, TTFields can interfere with the formation and directional movement of microtubulin,ultimately leading to the apoptosis of tumor cells).

TTfields disrupt the normal mitotic process by acting on key charged macromolecules or organelles during mitosis, thereby destroying cells and achieving the purpose of tumor suppression. Two basic physics principles are involved in this process: dipole alignment and dielectrophoresis. Under the action of a uniform alternating electric field, in order to maintain a safe parallel with the power vector, the charged molecule will oscillate continuously, and the positive and negative charges within the molecule will be separated, so that they will align themselves parallel to the direction of the exposed power vector. In order for the cell to properly mitosis, key macromolecules and organelles in the process of mitosis and cytoplasmic division are highly polarized, so that the charged structures in each stage of cell division are precisely aligned. Therefore, their random motion is disturbed by externally applied local electrical fields (43). Under the action of the TTfields electric field, the normal random movement of microtubule subunits in the cytoplasm during metaphase division is disturbed, resulting in the suspension of the normal microtubule assembly of the spindle, resulting in asymmetric chromatin separation. In normal division, the 2/6/7 aggregates were recruited to the midline of the spindle at a later stage, and under the action of the parallel distribution of cytofissure fibers, the cleavage groove evolved and gradually narrowed, with the boundary axis parallel to the direction of the applied alternating electric field. The TTfields interfere with this process by disrupting the ability of individual polymers to bind to each other, inhibiting the formation of cleavage proteins. In the absence of normal cleavage protein function, the contraction of dividing cells cannot be confined to the midline of the cell equator, resulting in severe contraction of the cell membrane and abnormal mitotic outlet at the beginning of the anaphase, and eventually a strong cytoplasmic blistering and cell membrane rupture. On the other hand, tubulin has a higher electric dipole moment (1660D),and the effect of TTfields on microtubules may be more significant due to the faster dynamic process of microtubule assembly relay. Therefore, under the action of TTfields, the dividing cells will show asymmetric chromatin separation, mitosis inhibition or division delay, which will lead to uneven distribution of chromosomes in daughter cells, and eventually cause cell stress. Stressed tumor cells induce host immune response under the influence of TTfields. In addition to disrupting cell division, TTfields can also interrupt DNA repair mechanisms (44–50) (**Figure 5**).

Therefore, TTfield has both direct and indirect anti-tumor mechanisms, and when the host immune system is involved at the same time, TTfield’s anti-tumor ability can reach the strongest. The abnormal structures of various tumor cells treated by TTfield were observed under immunofluorescence microscopy, including chromosome malarrangement in prepolyp, middle polyp, middle uniaxial and late polyp asymmetrical chromosome separation. These cellular phenomena are affected by the frequency of alternating electric fields, with an effective range of 100-300 KHZ and an optimal frequency of 200kHz. The optimal frequency of TTfield is related to cell size, which may be why the optimal frequency of mesothelioma cell lines, lung adenocarcinoma cells, and breast cancer differs from GBM.

### 5.2 Research progress of TTfields combined therapy in glioma

#### 5.2.1 Efficacy of TTFields-TMZ combined therapy for newly diagnosed GBM patients

To evaluate the efficacy and safety of TTFields combined with temozolomide(TMZ) maintenance therapy in GBM patients after chemoradiotherapy. Stuppa analyzed through 210 clinical patients, including 210 patients randomized to TTFields combined with TMZ and 105 patients randomized to TMZ alone, with a median follow-up of 38 months (range 18-60 months). Median progression-free survival was 7.1 months (95% CI, 5.9-8.2 months) in the TTFields group and 4.0 months (95% CI, 3.3-5.2 months) in the TMZ group alone (hazard ratio [HR], 0.62 [98.7% CI], 0.43-0.89]; *P* =.001). Median overall survival was 20.5 months (95% CI, 16.7-25.0 months) in the TTFields Gatimozolomide group (n=196) and 15.6 months (95% CI, 13.3-19.1 months) in the TMZ group alone (n= 84) (HR, 0.64 [99.4% CI, 0.42-0.98]; *P*=.004). Their study showed that the combination of TTFields with TMZ maintenance chemotherapy significantly extended progression-free survival and overall survival in an interim analysis of GBM patients with standard chemoradiotherapy. In addition, Fishman H’s study showed that TTField could improve the effectiveness of TMZ and Lomustine in GBM cell lines (51, 52).

#### 5.2.2 TTField is used to treat recurrent GBM

In 2007, Kirson and other research teams used TTField to treat relapsed glioma through 10 small-scale clinical trials, and the median PFS of patients reached 26.1 weeks, and the PFS at 6 months was 50%, and there were still 2 patients with no progression until the end of the study. The median overall survival was 62.2 weeks, much higher than the previous median tumor progression time of 9.5 weeks and median OS 29.3 weeks, confirming the efficacy of TTField in the treatment of recurrent glioma. Meanwhile, studies have also confirmed that TTField does not induce arrhythmia and seizures (53). At the same time, Roger Stuppa et al. conducted a phase III clinical trial of recurrent GBM, in which 237 patients with recurrent glioma were randomly divided into a single tumor electric field therapy group (n=120) and an optimized therapy group (n=117) in a 1:1 ratio. Chemotherapy group was the best choice for doctors. The results showed that the median overall survival was 6.6 months and 6.0 months (HR=0.86,*P*=0.27) in the electric field therapy group and the optimal treatment group, respectively. The 1-year survival rates were 20% and the median PFS were 2. Months and 2.1 months (HR=0.81, *P*=0.16), 21.4% and 15.1% progression-free surial was divided into 6 months (*P*=0.13), the imaging efficiency are 14% and 9.6% respectively (*P*=0.19). The living quality assessment showed that the symptoms of constipation, nausea and vomiting caused by tumor electric field therapy were significantly reduced compared with chemotherapy, and helped improve the cognitive function of patients. Although compared with traditional chemotherapy drugs, this trial failed to show that electric field therapy could improve the survival rate, its efficacy was comparable to that of traditional chemotherapy, and at the same time, it could significantly improve the quality of life, becoming a highlight of its treatment (54). Similarly, Mrugala analyzed data from 457 relapsed GBM patients treated with TTfield at 91 U.S. cancer centers and showed that patients treated with TTfields for relapsed GBM had a significant benefit (55).

To sum up, in clinical practice, the efficacy of TTfields is worthy of affirmation, and it has high patient tolerance and good safety, which is worthy of clinical promotion.

### 5.3 Effects of TTFields combined with radiotherapy or drugs on GBM molecular pathway

**Figure 6:**
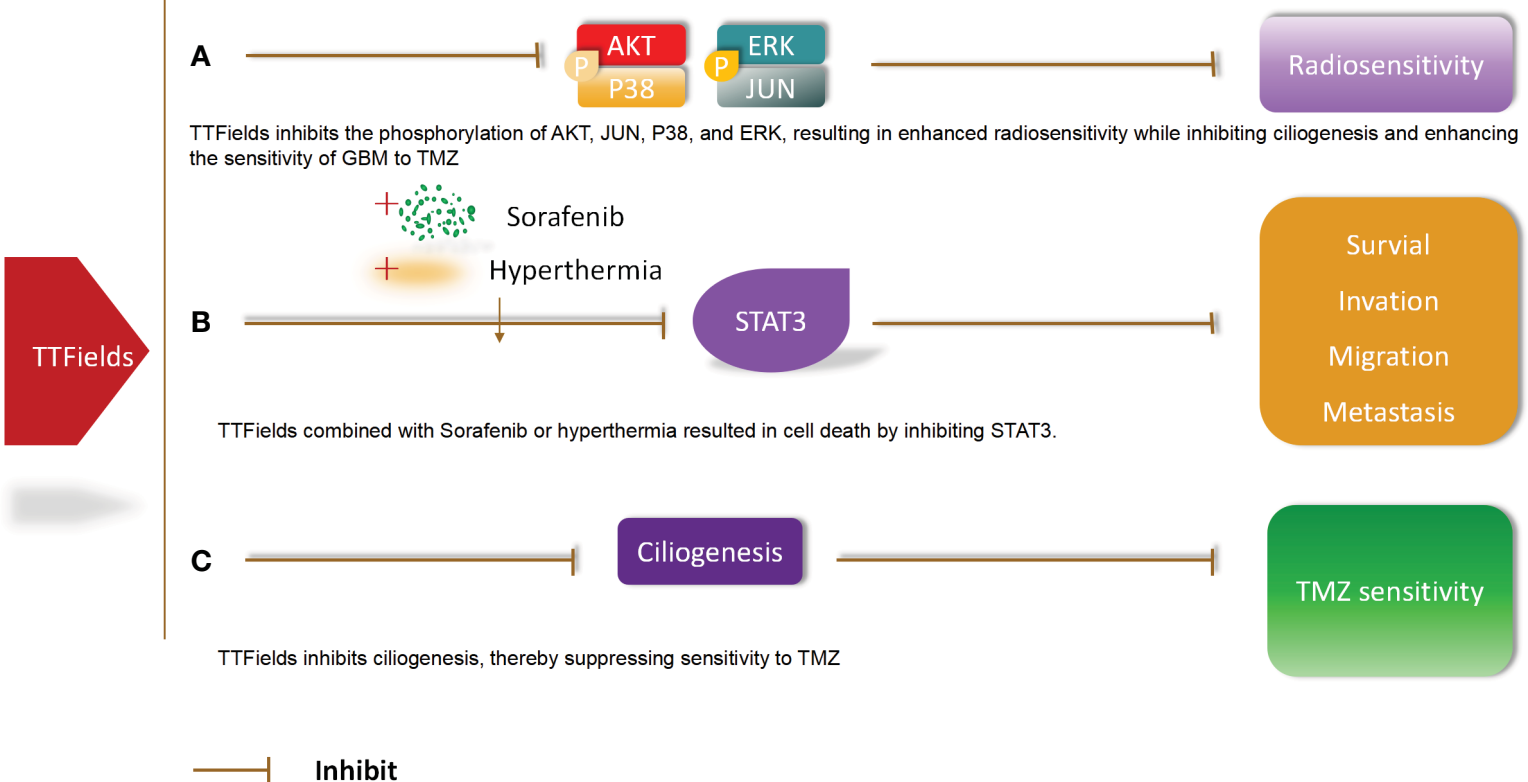
Molecular pathway changes caused by TTFields combined with radiotherapy or drugs in GBM.

Molecular pathway changes caused by TTFields combined with radiotherapy or drugs in GBM. TTFields inhibits the phosphorylation of AKT,JUN,P38,and ERK, resulting in enhanced radiosensitivity while inhibiting ciliogenesis and enhancing the sensitivity of GBM to TMZ. In addition, TTFields combined with Sorafenib or hyperthermia resulted in cell death by inhibiting STAT3.TTFields inhibits ciliogenesis, thereby suppressing sensitivity to TMZ (56) (**Figure 6**).

### 5.4 Molecular pathway changes caused by TTFields on glioma and GBM

**Figure 7:**
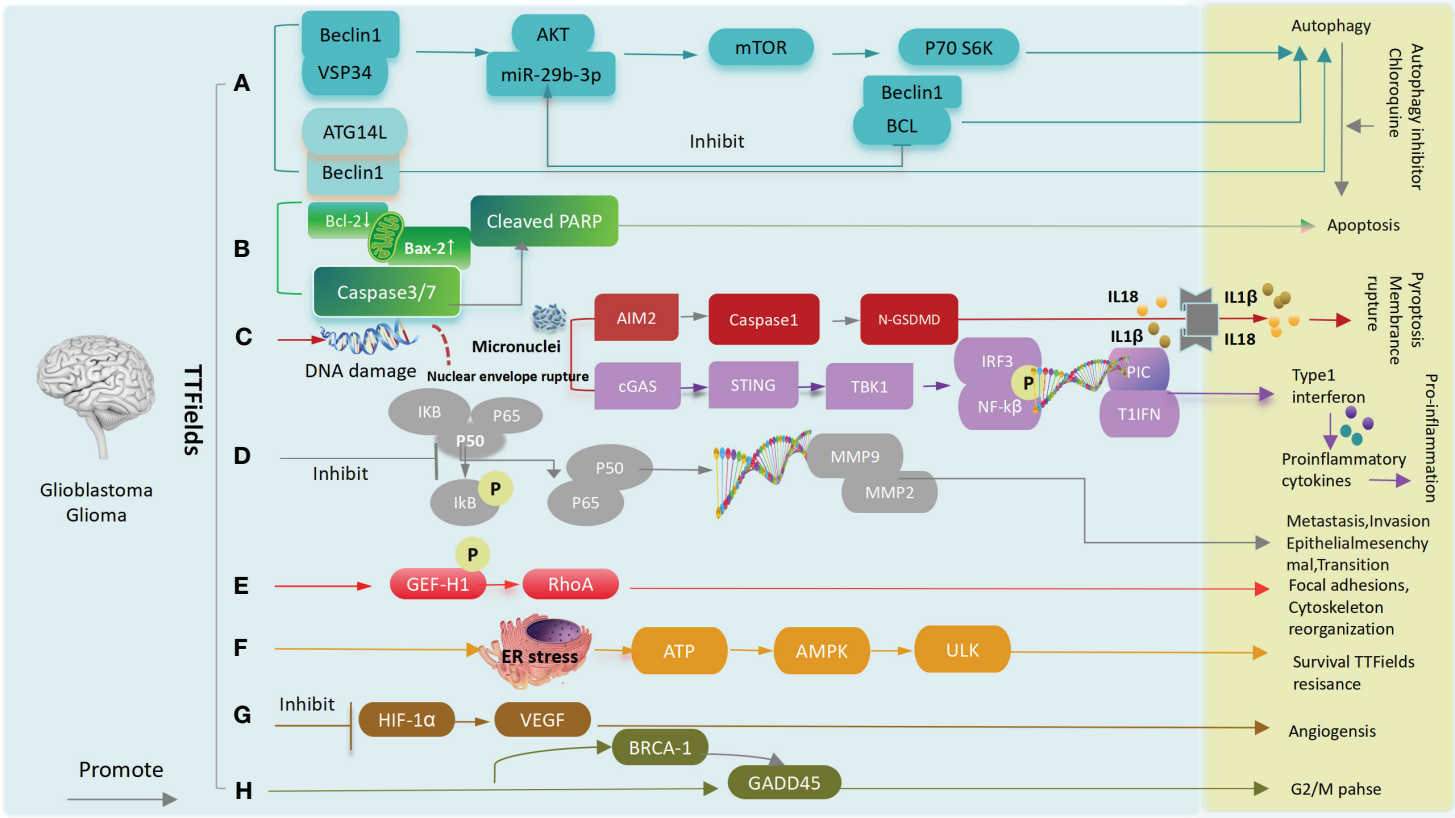
Molecular pathway changes caused by TTFields on glioma and GBM.

A:After TTFields treatment, Beclin1 increases the binding of Atg14L and Vps34(the positively regulated autophagosome) and decreases Bcl-2(the negatively regulated autophagosome),leading to glioma cells and tumor stem cell autophagy. Meanwhile, activation of the AKT2/mTOR/p70S6K axis also leads to autophagy. B:TTFields up-regulates caspase3, caspase7 or increases BAX, down-regulates BCL-2 expression, and leads to apoptosis. C:TTFields destroys the nuclear membrane, generates micronuclei and double strand breaks, activate the cGAS-Sting signaling pathway to increase the expression of proinflammatory factors and type I interferon, and through the AIM2-Caspase1 inflammasome Cleavage of GSDMD and release of LDH leads to pyroptosis and immune activation ultimately. D: TTFields inhibits IkBα phosphorylation and NF-kB p65 translocation, the expression of MMP2 and MMP9,and ultimately inhibits cell invasion, metastasis, and EMT processes. E: TTFields promotes phosphorylation of GEF-H1,which further activates RhoA, ultimately leading to focal adhesions and cytoskeleton reorganization. F:TTFields causes endoplasmic reticulum stress and releases ATP, which activates AMPK and ULK, leading to resistance to TTFields. G:TTFields attenuates tube formation and angiogenesis by down-regulating the expression of HIF1α and VEGF. H:Upregulation of BRCA1 and GADD45 results in G2/M phase arrest (45, 56–59) (**Figure 7**).

### 5.5 Limitations of TTfields treatment

At present, the most common adverse reaction to TTfields treatment is mild or moderate dermatitis of the skin at the site of electrode placement, which is likely due to a variety of factors, including persistent moisture, poor skin heat loss, chemical irritation of hydrogels and medical tape components, and TTfields may inhibit normal epithelial cell proliferation in the skin. Although most are mild and moderate injuries, which can usually be treated by moving the array 1-2cm or using cortisol locally, severe infections and ulcers can cause permanent damage to the patient. The high cost of TTFields treatment is one of the important factors limiting the adoption of this technology in the treatment of neurological tumors, and TTFields treatment is considered cost-benefit in the United States health care system, but national health care policies vary. Although researchers have explored the therapeutic mechanisms of TTFields, clinicians remain skeptical of the technology.

In conclusion, TTfields is a new approach to non-invasive cancer treatment. Clinically, its efficacy and safety have been demonstrated in the treatment of newly diagnosed and relapsed glioblastoma. TTfields is able to selectively kill rapidly dividing cells by interrupting cell division. As a result, TTfields can be applied to a wide range of local tumors, including GBM. In addition to further optimizing treatment options for GBM, TTfields has broad application prospects for treating other cancers.

## 6 Glioma therapy: new challenges and nanomaterial-based methods

**Figure 8:**
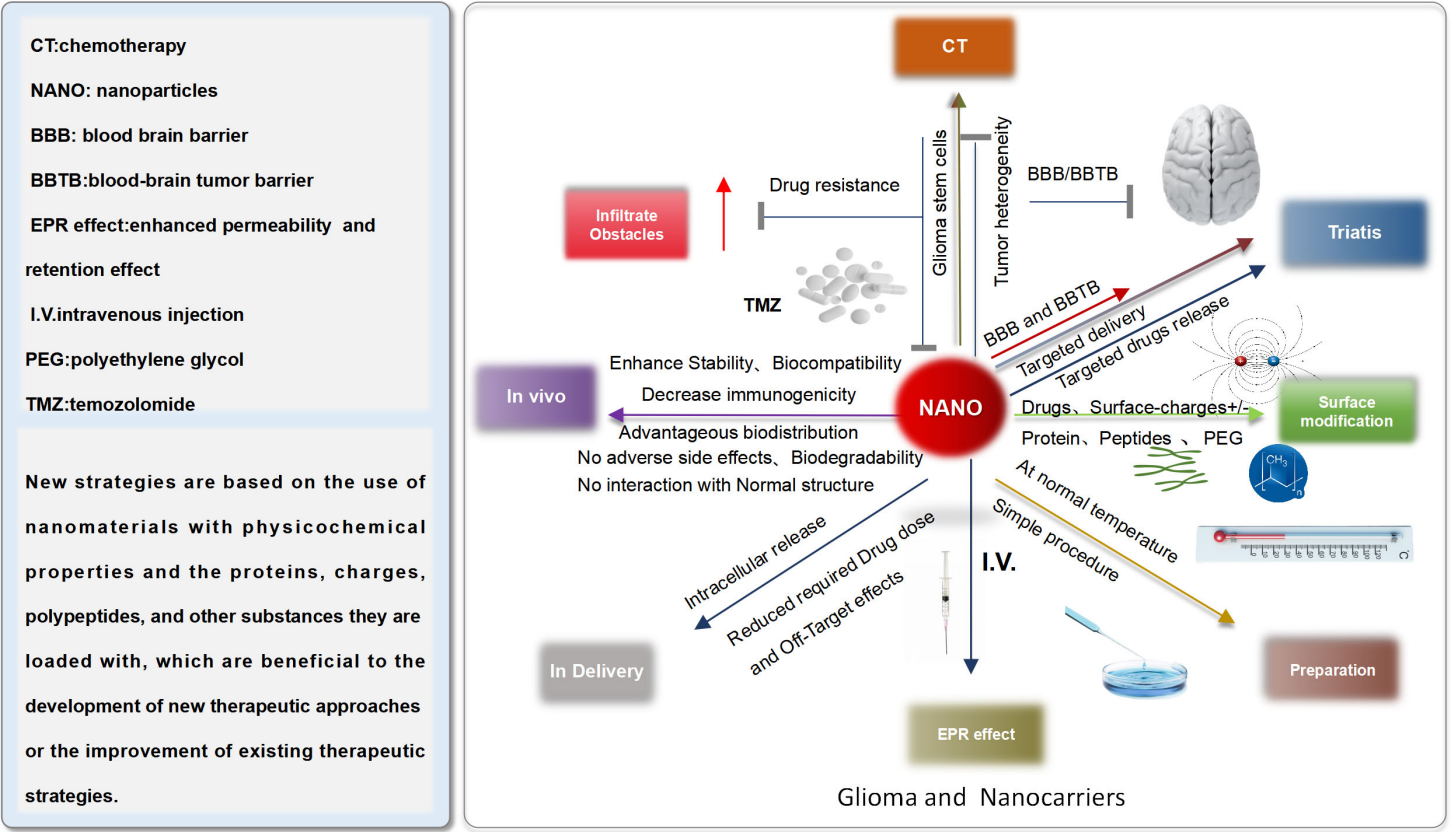
Characteristic of standard nanomaterial.

Although glioma therapy still remains a huge challenge for researchers and clinicians, the booming of nanotechnology (NT) provides potential approaches for prospective glioma treatment. However, Bloodbrain barrier (BBB), blood-brain tumor barrier(BTB), hypoxia, and complex tumor immune environment (TIE) hinder the development of the new medical technology. In order to address above items, researchers and clinicians, have been dedicated to designing diversified nanoformulations and standard nano drug delivery system for enhancing glioma therapeutic effect (**Figure 8**).

In spite of the limitations of BBB, some nanocarriers have been used to deliver chemotherapy drug for brain tumor therapy. NDDS can provide many preponderances, for instance 1) Enhancing the BBB penetration depth and bioavailability of the drugs in tumor tissues; 2) Targeted and controlled drug release or pH responsive release; 3) Multiple diverse drugs can be co-modified in the surface of nanocarriers, reaching the combined therapy;4)No or little toxicity (60).

At present, superparamagnetic iron oxide nanoparticles (SPIONs) represent the most widely used theranostic magnetic MNPs for various biomedical applications, such as high-contrast agents for MRI (60, 61), efficient drug delivery (62), and magnetism-based hyperthermia therapy (63). Various SPION-based formulations have been synthesized as functional nanoplatforms for imaging and therapy of brain tumors. Meanwhile, NIR fluorescent nanoprobes, gold nanomaterials, Micro/nanobubbles, Mesoporous silica NPs (MSNPs),Mesoporous ruthenium NPs (MRNs),and Titanium dioxide NPs have been used for diagnosis and therapy of glioma.

Nanotechnology provides the full potential to address GBM treatment. However, some challenges and continuing efforts are needed to accomplish the translation of glioma nanomedicine from fundamental research to clinic.

## 7 Discussion

At present, PDT, LITT and TTFields have been applied in the treatment of glioma, and PDT has been combined with surgery, and certain clinical effects have been achieved. Some studies have shown that the overall survival rate of patients within 12 months can reach 95.5%, but large sample studies are still needed to verify. LITT can achieve accurate positioning and real-time monitoring of gliomas, and maximize the protection of non-tumor tissues. For gliomas with recurrence, deep location, unsuitable for surgical treatment or ineffective standard treatment, LITT is considered as a potential local treatment method, which can effectively improve the prognosis of patients and living quality. It can avoid the risks associated with craniotomy to remove tumors, while reducing hospital stays and cost of hospitalization, but the long-term effectiveness of this treatment still needs to be evaluated through rigorous randomized clinical trials. The most successful application so far is TTFields, which has entered clinical translation. However, the clinical application of other minimally invasive treatments is limited due to natural barriers such as skull and blood-brain barrier. However, these programs can be used as auxiliary means to gradually carry out by selecting appropriate cases at the bedside, but their safety and effectiveness still need to be further verified.

## 8 Conclusion and future perspectives

In recent years, with the continuous development of medical technology, minimally invasive technology of nervous system tumor is constantly improving. Minimally invasive surgery using neural navigation system combined with robotic technology has become an important method in the treatment of various diseases in neurosurgery, which significantly improves the precise positioning of surgery and the thoroughness of tumor resection. In addition, the emergence of new technologies such as NDDS, immunotherapy, gene therapy and cell therapy has also provided more options and applications for minimally invasive treatment of glioma. Although there are still some limitations and challenges, its therapeutic effect is still worthy of recognition and promotion in clinical practice. Future research directions include further improving minimally invasive surgical techniques and endovascular intervention techniques, exploring novel, effective and safe therapeutic approaches, optimizing multimodal treatment strategies, and exploring individualized treatment options to further extend patient survival and improve living quality of patients.

## Author contributions

HW: Writing – original draft, Writing – review & editing. FZ: Writing – original draft. WG: Data curation, Methodology, Writing – original draft. PC: Investigation, Software, Writing – review & editing. YW: Formal Analysis, Writing – review & editing. FW: Resources, Supervision, Writing – original draft. HZ: Funding acquisition, Supervision, Writing – original draft.
